# The Efficacy and Safety of Mesalamine and Probiotics in Mild-to-Moderate Ulcerative Colitis: A Systematic Review and Meta-Analysis

**DOI:** 10.1155/2020/6923609

**Published:** 2020-03-28

**Authors:** Chunying Tian, Yang Huang, Xiaoxia Wu, Chuhan Xu, Huaien Bu, Hongwu Wang

**Affiliations:** Tianjin University of Traditional Chinese Medicine, Tianjin 301617, China

## Abstract

**Objective:**

To evaluate the efficacy and safety of mesalamine in conjunction with probiotics for ulcerative colitis.

**Methods:**

Random controlled trials (RCTs) were searched in PubMed, EMBASE, Cochrane Library, China National Knowledge Infrastructure, Wanfang, and VIP (VIP Database for Chinese Technical Periodicals) from inception to October 2019. Methodological quality was assessed by the Cochrane Collaboration tool. The quality of evidence was rated by the Grading of Recommendations, Assessment, Development, and Evaluation (GRADE). Data analysis was carried out in Review Manager 5.3.

**Results:**

A total of fifteen studies met the criteria for inclusion. Thirteen studies reported the clinical efficacy, three studies provided data on the clinical symptom scores, two trials reported disease activity index, four studies evaluated endoscopic score, and twelve studies reported adverse events. For ulcerative colitis (UC), mesalamine and probiotics had better clinical efficacy than mesalamine alone (≤8 weeks: RR = 1.12, 95% CI: 1.07–1.18, *P* < 0.0001; >8 weeks: RR = 1.25, 95% CI: 1.11–1.41, *P*=0.0003). On the clinical symptom scores, disease activity index, and endoscopic score, UC patients receiving mesalamine and probiotics had significant difference than patients receiving mesalazine alone (MD = −2.02, 95% CI: −3.28 to −0.76, *P*=0.002; MD = −1.20, 95% CI: −1.76 to −0.65, *P* < 0.001; and MD = −0.42, 95% CI: −0.61 to −0.23, *P* < 0.0001, respectively). There was no statistically significant difference in adverse events between the two groups (RR = 0.88, 95% CI: 0.54 to 1.43, *P*=0.60).

**Conclusion:**

Our meta-analysis results supported that mesalamine and probiotics were effective and safe in treating ulcerative colitis.

## 1. Introduction

Ulcerative colitis (UC) is a chronic inflammatory bowel disease (IBD) with onset most frequently in adults aged 30–40 years [1]. The worldwide incidence and prevalence of UC have been increasing over the last few decades [2]. The highest annual incidence of UC was 24.3 per 100,000 person-years in Europe, 6.3 per 100,000 person-years in Asia and the Middle East, and 19.2 per 100,000 person-years in North America [3]. Most patients with UC have a mild-to-moderate course characterized by suffering from a relapsing and remitting course [4, 5]. For the American Gastroenterological Association institute guideline on the management of mild-to-moderate ulcerative colitis [5], mild-to-moderate UC was defined as patients with <4–6 bowel movements per day, mild-to-moderate rectal bleeding, absence of constitutional symptoms, low overall inflammatory burden, and absence of features suggestive of high inflammatory activity, based on Truelove and Witt's criteria [6] and the Mayo Clinic score [7]. Although the etiology of UC remains unclear, an excessive immune response to endogenous bacteria in genetically predisposed individuals may play an important role in the pathophysiology of UC [8–10].

The existing mainstay of therapy for mild-to-moderate UC is the 5-ASA class of medications, including mesalamine, sulfasalazine, and diazo-bonded 5-ASA [11]. Mesalamine is a first-line treatment for many patients with UC. It gives anti-inflammatory effects by increasing expression of peroxisome proliferator-activated receptors in gastrointestinal epithelial cells. Furthermore, it acts to inhibit COX enzymes, thus affecting prostaglandins and decreasing inflammation of the colon [12–16]. Unfortunately, it is difficult to cure UC completely, with 74% of patients experiencing at least one relapse during 5-year observation in a prospective population-based cohort study [17]. Furthermore, taking these drugs could lead to the occurrence of various adverse effects [18]. Therefore, new therapeutic optimizations are required in order to improve clinical efficacy.

However, UC is characterized by periods of activity and UC patients can experience frequent relapses [5]. After the imbalance of gastrointestinal flora, the decrease of the number of beneficial bacteria such as caseating bacteria causes the patients' intestinal epithelial cells' lack of energy source, and the increase of harmful bacteria and pathogenic bacteria in the intestinal tract will cause hydrolytic protein and enterotoxin, which will damage the patients' intestinal mucosal cells, activate immune cells, increase intestinal mucosal permeability, and induce immune response [19]. Therefore, the treatment that is directly modulating the gut microbiota is an attractive therapeutic option for UC [20]. Probiotics contain viable microorganisms, sufficient amounts of which reach the intestine in an active state for them to exert positive health effects [21]. They mostly include lactic acid-producing bacteria, such as bifidobacteria and lactobacilli. Many studies showed that these probiotics modulated membrane permeability and the mucosal immune system [20, 22]. However, the sample size was relatively small, such that there was no definitive evidence as to whether probiotics were helpful. Thus, we performed a systematic review and meta-analysis based on a large data of RCTs, making the evidence more convincing.

## 2. Materials and Methods

### 2.1. Search Strategy

We performed a comprehensive literature search in the PubMed, EMBASE, Cochrane Library, China National Knowledge Infrastructure, Wanfang, and VIP (VIP Database for Chinese Technical Periodicals) from inception to October 2019. The search terms used are as follows: mesalazine, mesalamine, probiotics, and ulcerative colitis. Reference list of included studies was also checked to identify potentially eligible studies. The identified studies were not constrained by language. Taking PubMed as an example, detailed search strategy was as follows:

  #1 “mesalamine” [MeSH Terms] OR “mesalamine” [Title/Abstract] OR “mesalazine” [Title/Abstract]
  #2 “probiotics” [MeSH Terms] OR “probiotics” [Title/Abstract]
  #3 “colitis, ulcerative” [MeSH Terms] OR “colitis, ulcerative” [Title/Abstract]
  #4 #1 AND #2 AND #3

### 2.2. Inclusion Criteria

Trials were selected based on the following inclusion criteria: (1) RCTs comparing mesalamine and probiotics with mesalamine alone; (2) patients diagnosed definitely as mild-to-moderate ulcerative colitis with no limits on their sex, age, or case resources; (3) trials reporting the clinical efficacy, the clinical symptom scores, disease activity index (DAI), endoscopic score, or adverse events as outcome. The criteria of clinical efficacy were as follows: effectiveness meant that the clinical symptoms disappeared basically and endoscopy showed normal or mild inflammation; ineffectiveness meant that there was no change in the clinical symptoms and endoscopy after treatment.

And exclusion criteria were (1) non-mild-to-moderate patients; (2) noninterventional studies, such as traditional review, systematic review and meta-analysis, case reports, cohort study, guideline or recommendation; (3) nonclinical studies, such as animal studies, theoretical research; (4) ongoing trial; (5) irrelevant studies; (6) repeated studies; and (7) studies with only abstract.

### 2.3. Data Extraction and Assessment of Quality

Two researchers (Chunying Tian and Yang Huang) independently performed the data extraction in a standard excel sheet. When disagreement was evident, a third researcher (Hongwu Wang) resolved the issue. For each study, the following information was extracted: the first author, publication year, study design, study location, sex, age, the course of disease, sample size, the dosage of treatment, treatment period, and outcome.

The methodological quality of included studies was assessed independently by two researchers (Chunying Tian and Xiaoxia Wu) based on the Cochrane Collaboration tool with the following domains: (1) generation of random sequence; (2) allocation concealment; (3) blinding of participants and personnel; (4) blinding of outcome assessment; (5) selective reporting; (6) incomplete outcome data; and (7) other bias. The results were assessed as high risk, unclear risk, and low risk. Besides, the Grading of Recommendations, Assessment, Development, and Evaluation (GRADE) [23] was used to rate the quality of evidence by the consensus of two authors (Chunying Tian and Huaien Bu).

### 2.4. Statistical Analysis

Statistical analysis was carried out in the Review Manager 5.3 (RevMan 5.3). Continuous data were expressed as mean difference (MD) with 95% confidence interval (CI), and the relative risk (RR) with 95% CI was calculated in the dichotomous data [24]. Heterogeneity of included data was evaluated with the use of the chi-square test and *I*^2^ statistic. The DerSimonian and Laird random-effects model was used when high heterogeneity was assessed (*I*^2^ > 50% or *P* < 0.10). Otherwise, the Mantel–Haenszel fixed-effects model was used for statistical analysis [25, 26]. Sensitivity analysis was conducted to assess the stability of pooled results. Publication bias was observed by an inverted funnel plot [27].

## 3. Results

### 3.1. Screening Process

**Figure 1:**
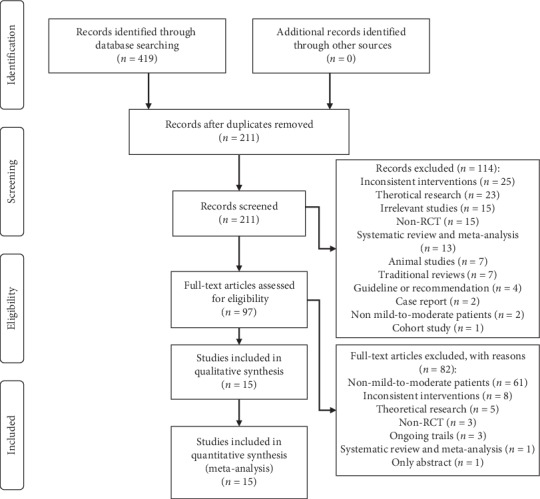
The flow chart of study selection.

A total of 419 related studies were initially retrieved from six databases, of which 208 were excluded due to duplication. Titles and abstracts of 211 studies were screened for inclusion. Full texts of 97 studies were read, and 15 studies [19, 28–41] met our inclusion criteria. The flow chart of study selection is presented in Figure 1.

### 3.2. General Characteristics of Included Studies

The included studies were published from 2008 to 2019. The sample size ranged from 34 to 360, with a total of 1433 patients. Fifteen studies involved four different ways of probiotics, including Bifid Triple Viable Capsules, *Bifidobacterium* TriPlex, *Lactobacillus* and *Bifidobacterium* quadruple viable tablet. The treatment period of fifteen studies ranged from 4 to 24 weeks. Thirteen reports [19, 28–30, 32–38, 40, 41] evaluated the clinical efficacy. Three studies [33, 36, 39] provided data on the clinical symptom scores. Four trials [33, 36, 39, 41] reported disease activity index. Two trials [36, 39] estimated endoscopic score. Twelve studies [19, 28, 29, 31–33, 35–40] reported adverse events. General characteristics of included fifteen studies are summarized in Table 1.

### 3.3. Assessment of Quality

**Figure 2:**
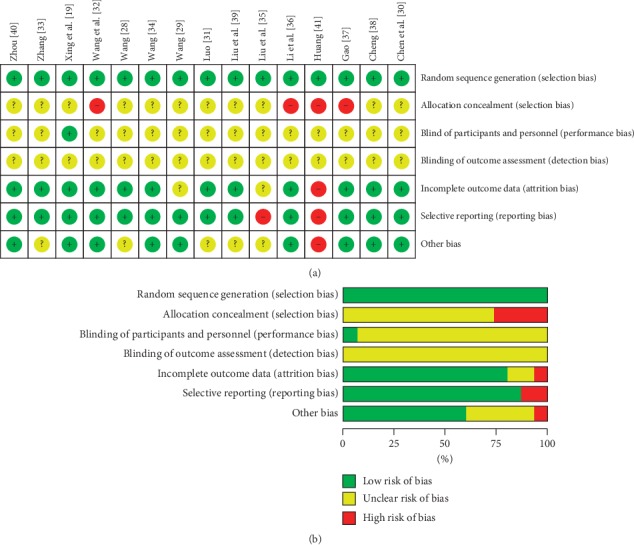
Risk of bias assessment using the Cochrane Collaboration tool. (a) Risk of bias summary. (b) Risk of bias graph.

All studies mentioned randomization and were rated as low risk of bias for this item. In assessing the risk of allocation concealment, four studies [29, 36, 37, 41] using random number table method were rated as high risk of bias for this item. Blindness was unclear in most studies. Only one study [19] mentioned single blindness and was assessed as low risk of bias. As to incomplete outcome data, one study [41] was rated as high risk of bias because there was missing data in results. Two studies [35, 41] were assessed as high risk of bias for selective reporting. The baseline of one study [41] was imbalanced. Therefore, it was rated as high risk of bias in other bias. The risk of bias assessment in the studies is presented in Figure 2.

### 3.4. Pooled Results

#### 3.4.1. Clinical Efficacy

**Figure 3:**
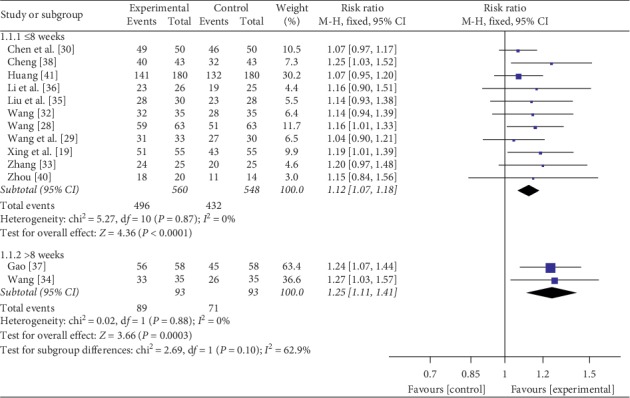
Forest plot of the clinical efficiency.

Thirteen studies [19, 28–30, 32–38, 40, 41] evaluated the clinical efficacy, which involved a total of 1294 patients. There was no heterogeneity between experimental group and control group when the course of treatment was less than 8 weeks (*P* = 0.87, *I*^2^ = 0%); thus, a fixed-effects model was used. Eleven studies [19, 28–30, 32, 33, 35, 36, 38, 40, 41] found that mesalamine and probiotics were superior to mesalamine alone on the clinical efficacy within 8 weeks (RR = 1.12, 95% CI: 1.07 to 1.18, *P* < 0.0001). And there was no statistical heterogeneity in the results when the course of treatment was more than 8 weeks (*P* = 0.88, *I*^2^ = 0%); thus, a fixed-effects model was used. Two studies [34, 37] also found that mesalamine and probiotics were superior to mesalamine alone on the clinical efficacy more than 8 weeks (RR = 1.25, 95% CI: 1.11 to 1.41, *P* = 0.0003) (Figure 3).

#### 3.4.2. Clinical Symptom Scores

**Figure 4:**
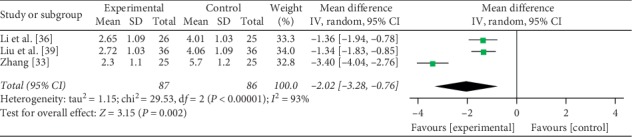
Forest plot of the clinical symptom scores.

Three studies [33, 36, 39] totaling 173 patients provided data on the clinical symptom scores. Among the 173 patients, mesalamine and probiotics were used as an experimental therapy in 87 patients, and 86 patients were treated by mesalamine. There was significant heterogeneity between experimental group and control group (*P* < 0.00001, *I*^2^ = 93%); thus, a random-effects model was used. Compared with mesalamine alone, the combination with probiotics was significantly different on the clinical symptom scores (MD = −2.02, 95% CI: −3.28 to −0.76, *P*=0.002) (Figure 4). After excluding one study (Zhang [33]), there was no heterogeneity (*P*=0.96, *I*^2^ = 0%); thus, a fixed-effects model was used. Sensitivity analysis showed that mesalamine and probiotics were significantly different on the clinical symptom scores compared with mesalamine alone (MD = −1.35, 95% CI: −1.72 to −0.97, *P* < 0.00001). And the results of the clinical symptom scores were stable.

#### 3.4.3. Disease Activity Index (DAI)

**Figure 5:**
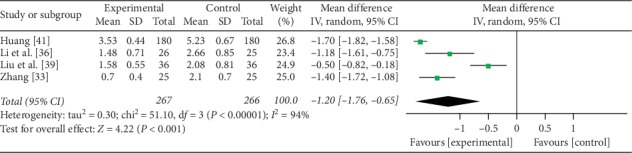
Forest plot of disease activity index.

Four trials [33, 36, 39, 41] reported disease activity index, which involved a total of 533 UC patients, 267 of them used mesalamine and probiotics for therapy, and 266 patients were treated by mesalamine. After performing a meta-analysis, there was significant heterogeneity between experimental group and control group (*P* < 0.00001, *I*^2^ = 94%); thus, a random-effects model was used. The result showed that there was significant difference between the two groups (MD = −1.20, 95% CI: −1.76 to −0.65, *P* < 0.001) (Figure 5). After excluding studies (Huang [41] and Liu et al. [39]), there was no heterogeneity (*P* = 0.42, *I*^2^ = 0%); thus, a fixed-effects model was used. Sensitivity analysis indicated that mesalamine and probiotics were significantly different on the DAI compared with mesalamine alone (MD = −1.32, 95% CI: −1.58 to −1.07, *P* < 0.00001). And the results of the DAI were stable.

#### 3.4.4. Endoscopic Score

**Figure 6:**
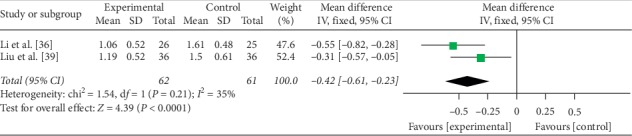
Forest plot of the endoscopic score.

Two trials [36, 39] involving 123 UC patients with probiotics in the adjuvant therapy analyzed the endoscopic score. There was no statistically significant heterogeneity (*P* = 0.21, *I*^2^ = 35%); thus, a fixed-effects model was used. Significant differences in UC patients were observed between the two groups, suggesting that the efficacy of probiotics in the adjuvant therapy of UC in the experimental group may be better than that in the control group (MD = −0.42, 95% CI: −0.61 to −0.23, *P* < 0.0001) (Figure 6).

#### 3.4.5. Adverse Events

**Figure 7:**
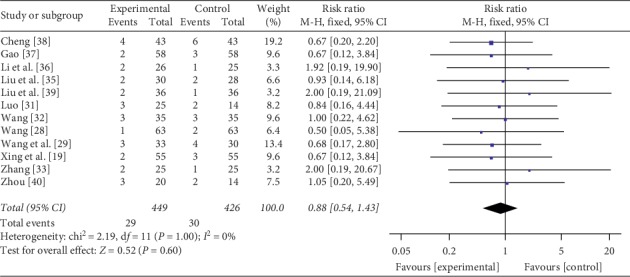
Forest plot of adverse events.

Twelve trials [19, 28, 29, 31–33, 35–40] with 875 participants reported adverse events. There was no heterogeneity in the results (*P*=1.00, *I*^2^ = 0%); thus, a fixed-effects model was used. Statistically significant difference was not found in the pooling of data between two groups (RR = 0.88, 95% CI: 0.54–1.43, *P*=0.60), suggesting that the safety of mesalamine and probiotics for UC was similar with mesalamine alone (Figure 7).

#### 3.4.6. Publication Bias

**Figure 8:**
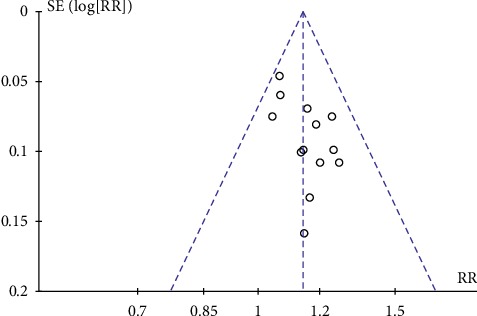
Funnel plot of publication bias.

There was an uneven distribution and light asymmetry in the funnel plot, which meant publication bias in the clinical efficacy between the experimental group and the control group may exist. The funnel plot is presented in Figure 8.

#### 3.4.7. Quality of the Evidence

**Figure 9:**
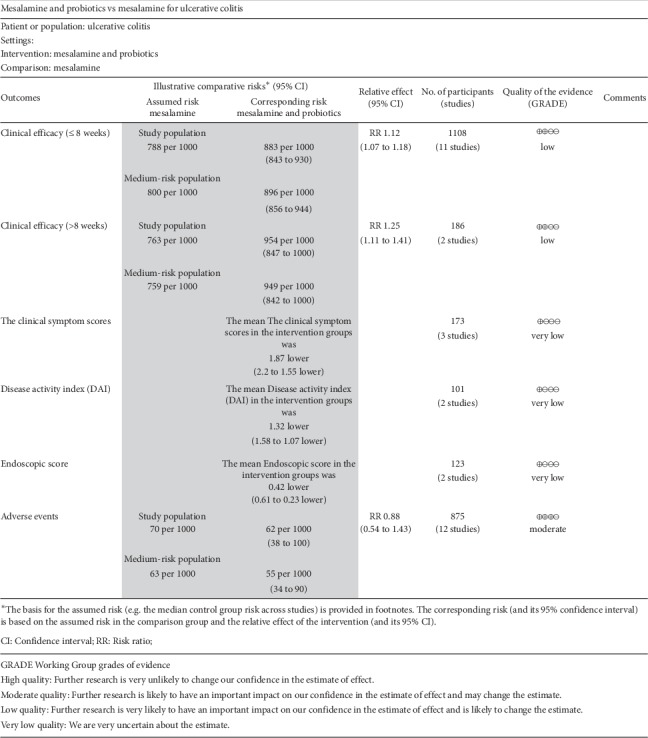
The Summary of findings.

The GRADE system was used to assess the quality of the evidence. In comparing the efficacy of mesalamine and probiotics with mesalamine, the quality of evidence is low in the outcome of the clinical efficacy. This is due to serious imprecision and reporting bias. The quality of evidence is very low in the three outcomes: the clinical symptom scores, DAI, and endoscopic score. This is due to the risk of bias, serious inconsistency, and serious imprecision. For the outcome “adverse events,” the quality of evidence is moderate because of the risk of bias. The summary of findings is presented in Figure 9.

## 4. Discussion

Continued improvements in the management of UC are needed to enable the most effective treatment possible for this disease. This is particularly important for UC of a mild-to-moderate severity, as the prevalence and incidence of UC are increasing worldwide [42]. The pathological and physiological mechanism of UC was complicated. It was reported that the intestinal flora for most patients with UC had changed, compared with normal healthy people [43]. The number of *Bifidobacterium*, *Helicobacter spiralis*, and *Pseudomonas* had decreased, while the number of *Enterococcus* and *Bacillus* increased. Therefore, correcting the imbalance of bacterial flora through probiotics is a possible treatment [44]. Many studies indicated that the mechanism of probiotics for UC patients was unclear, and the effect of mesalamine was obvious [45]. The possible mechanisms of probiotics in the therapy of UC mainly include the following: (1) to prevent pathogen infiltration by restraining bacterial adherence and bacteria translocation, or to produce antibacterial substances that inhibit the growth pathogenic bacteria; (2) to improve the function of epithelial mucosal barter; (3) to regulate the mucosal immune response; and (4) to reduce the secretion of proinflammatory factors [46]. Therefore, if probiotics was used when mesalamine was taken, the treatment plan would regulate the intestinal flora, curb the inflammatory responses, and improve therapeutic efficacy [46]. Furthermore, the advantages of probiotics therapy include overcoming gastrointestinal tract infections, resisting stomach acid, bile, and antibiotics, and modifying immune processes to destroy invading microorganisms [47].

The present study comprehensively and systematically reviewed the current available literature and found the following: (1) For mild-to-moderate UC patients, mesalamine in conjunction with probiotics had more significant therapeutic potential than mesalamine alone on the clinical efficacy no matter the treatment period was less than eight weeks or more, which indicated it could improve the clinical efficacy for mild-to-moderate UC patients. (2) Mesalamine and probiotics had significant difference for UC patients on the clinical symptom scores than mesalamine alone, which showed that clinical symptom scores were decreased and the clinical symptoms were alleviated, such as diarrhea, mucosa bloody stool, and abdominal pain. (3) Patients who took both mesalamine and probiotics had better results on the DAI than those who took mesalamine alone, which meant that the DAI was decreased and the frequency of diarrhea, mucosal manifestations, and bleeding was relieved. (4) For UC patients, significant differences on the endoscopic score were observed between the experimental group and the control group, which demonstrated that the condition of mucosa under endoscopy was improved. (5) Adverse events reported in these studies included nausea, abdominal extension, abdominal discomfort, headache, rash, fever, and liver dysfunction. The safety of mesalamine and probiotics for UC was similar to mesalamine alone, suggesting that mesalamine and probiotics did not increase the risk of drug use and had high safety. (6) In the quality assessment, the included studies were generally low risk of bias. And funnel plot showed there was light publication bias. (7) According to the GRADE assessment, the clinical efficacy was judged as low quality of evidence, the clinical symptom scores, DAI and endoscopic score were judged as very low quality of evidence, and adverse event was judged as moderate quality of evidence. Therefore, clinicians need to consider comprehensively when mesalamine and probiotics are used for mild-to-moderate ulcerative colitis patients, due to the complexity of clinical decision-making.

This review had certain limitations which were worthy of consideration. Primarily, there were no specific descriptions of generation of random sequence, allocation concealment, and blindness in most studies. Therefore, methodological limitations and the small sample size reduced the reliability of results. In addition, rating results of GRADE tool showed that three outcomes were rated as very low quality of the evidence. Two outcomes were, respectively, rated as low and moderate quality of the evidence. Thus, more well-designed and large-scale studies are warranted. Besides, there was a visible heterogeneity on the clinical symptom scores, DAI, and endoscopic score. According to the sensitivity analysis, the possible explanations about heterogeneity were presented as follows: the baseline difference of the included studies and the small sample size may contribute to the heterogeneity. Finally, the patients of included studies were Chinese, which limited generalizability of findings and caused publication bias. In fact, the inverted funnel plot indicated that there was a light publication bias, which may influence the stability of results.

## 5. Conclusion

In conclusion, our meta-analysis results supported that mesalamine and probiotics were effective and safe treatment for ulcerative colitis. Although the treatment plan appears to be effective and safe in this meta-analysis, further well-designed, more rigorous, and standardized studies are needed to confirm our results.

## Figures and Tables

**Table 1:** General characteristics of included studies.

Author, Publication year	Study design	Study location	Sex (M/F)	Age (year) (mean ± SD) (or range)	The course of disease (mean ± SD) (or range)	Sample size	The dosage of treatment		Treatment period (week)	Outcome
							T	C		
Wang 2018 [28]	RCT	China	T: 32/31 C: 33/30	T: 32.49 ± 3.76 C: 32.61 ± 4.04	T: 16.75 ± 4.46 m C: 16.94 ± 4.68 m	126	Mesalamine 0.5 g, TID + Bifid Triple Viable Capsules 3 capsules, BID	Mesalamine 0.5 g, TID	4	①⑤
Wang et al. 2013 [29]	RCT	China	T: 19/14 C: 19/11	—	T: 0.5∼5.5*y* C: 0.5∼6*y*	63	Mesalamine 1.5 g, TID + Bifid Triple Viable Capsules 2 capsules, TID	Mesalamine 1.5 g, TID	8	①⑤
Chen et al. 2019 [30]	RCT	China	T: 25/25 C: 28/22	T: 69.26 ± 9.30 C: 69.11 ± 9.14	T: 3.41 ± 2.37*y* C: 3.63 ± 2.50*y*	100	Mesalamine 1.0 g, QID + Bifidobacterium triplex 0.42 g, TID	Mesalamine 1.0 g, QID	8	①
Luo 2008 [31]	RCT	China	T: 25/14 C: 19/9	T: 19∼59 C: 21∼69	T: 0.5∼13*y* C: 0.5∼11*y*	67	Mesalamine 0.5∼1.0 g, TID + Bifid Triple Viable Capsules 0.42 g, TID	Mesalamine 0.5∼1.0 g, TID	8	⑤
Wang 2013 [32]	RCT	China	—	T: 20∼75 C: 17∼72	T: 0.5∼5.5*y* C: 0.5∼6*y*	70	Mesalamine 1.0 g, QID + combined Bifidobacterium and Lactobacillus 2 g, TID	Mesalamine 1.0 g, QID	4	①⑤
Zhang 2017 [33]	RCT	China	T: 15/10 C: 14/11	T: 42.3 ± 2.1 C: 44.9 ± 3.7	—	50	Mesalamine 1.0 g, QID + Bifidobacterium triplex 0.42 g, TID	Mesalamine 1.0 g, QID	8	①②③⑤
Wang 2016 [34]	RCT	China	T: 19/16 C: 21/14	T: 45.3 ± 9.7 C: 44.6 ± 9.4	T: 5.3 ± 1.5*y* C: 4.8 ± 1.2*y*	70	Mesalamine 1.0 g, TID + Lactobacillus 100 ml, TID	Mesalamine 1.0 g, TID	24	①
Xing et al. 2017 [19]	RCT	China	T: 29/26 C: 28/27	T: 34.0 ± 5.0 C: 33.4 ± 4.5	T: 4.5 ± 0.5*y* C: 4.0 ± 0.5*y*	110	Mesalamine 1.0 g, QID + Bifid Triple Viable Capsules, 2∼4 capsule, BID	Mesalamine 1.0 g, QID	8	①⑤
Liu et al. 2010 [35]	RCT	China	—	—	—	58	Mesalamine 1.0 g, QID + Bifid Triple Viable Capsules 0.42 g, TID	Mesalamine 1.0 g, QID	4	①⑤
Li et al. 2017 [36]	RCT	China	T: 14/12 C: 13/12	T: 43.2 ± 5.1 C: 42.6 ± 4.9	—	51	Mesalamine 1.0 g, QID + Bifidobacterium triplex 0.42 g, TID	Mesalamine 1.0 g, QID	8	①②③④⑤
Gao 2018 [37]	RCT	China	T: 33/25 C: 32/26	T: 35.26 ± 6.26 C: 35.67 ± 6.14	T: 5.52 ± 2.02*y* C: 5.74 ± 1.68*y*	116	Mesalamine 1.0 g, TID + Bifid Triple Viable Capsules 0.42 g, TID	Mesalamine 1.0 g, TID	12	①⑤
Cheng 2018 [38]	RCT	China	T: 22/21 C: 24/19	T: 39.5 ± 3.3 C: 38.4 ± 3.2	T: 3.6 ± 0.5*y* C: 3.5 ± 0.8*y*	86	Mesalamine 1.0 g, TID + Bifidobacterium quadruple viable tablet 0.2 g, TID	Mesalamine 1.0 g, TID	8	①⑤
Liu et al. 2013 [39]	RCT	China	T: 16/20 C: 14/22	T: 49.36 ± 2.62 C: 52.28 ± 2.53	—	72	Mesalamine 1.0 g, QID + Bifidobacterium triplex 0.42 g, TID	Mesalamine 1.0 g, QID	8	②③④⑤
Zhou 2019 [40]	RCT	China	T: 13/7 C: 9/5	T: 19∼56 C: 21∼61	T: 0.5∼5*y* C: 0.5∼12*y*	34	Mesalamine 1.0 g, TID + Bifidobacterium triplex 0.42 g, TID	Mesalamine 1.0 g, TID	8	①⑤
Huang et al. 2018 [41]	RCT	China	T: 90/90 C: 81/99	T: 42.2 ± 9.4 C: 41.5 ± 8.3	T: 5.5 ± 1.8*y* C: 5.2 ± 1.7*y*	360	Mesalamine 1.0 g, TID + Bifid Triple Viable Capsules 0.42 g, TID	Mesalamine 1.0 g, TID	8	①③

*Note*. —: not mentioned; ①: clinical efficacy; ②: the clinical symptom scores; ③: disease activity index (DAI); ④: endoscopic score; and ⑤: adverse events.

## Data Availability

This paper contains data that support the results of this study.
